# Down-regulation of promoter methylation level of *CD4* gene after MDV infection in MD-susceptible chicken line

**DOI:** 10.1186/1753-6561-5-S4-S7

**Published:** 2011-06-03

**Authors:** Juan Luo, Ying Yu, Huanmin Zhang, Fei Tian, Shuang Chang, Hans H Cheng, Jiuzhou Song

**Affiliations:** 1 Animal & Avian Sciences Department, University of Maryland, College Park, Maryland, 20740, USA; 2 USDA, ARS, Avian Disease and Oncology Laboratory, East Lansing, MI 48823, USA; 3 College of Animal Sciences, China Agricultural University, Haidian, Beijing, 100193, P.R. China

## Abstract

**Background:**

Marek’s disease virus (MDV) is an oncovirus that induces lymphoid tumors in susceptible chickens, and may affect the epigenetic stability of the *CD4* gene. The purpose of this study was to find the effect of MDV infection on DNA methylation status of the *CD4* gene differed between MD-resistant (L6_3_) and –susceptible (L7_2_) chicken lines.

**Methods:**

Chickens from each line were divided into two groups with one group infected by MDV and the other group as uninfected controls. Then, promoter DNA methylation levels of the *CD4* gene were measured by Pyrosequencing; and gene expression analysis was performed by quantitative PCR.

**Results:**

Promoter methylation of the *CD4* gene was found to be down-regulated in L7_2_ chickens only after MDV infection. The methylation down-regulation of the *CD4* promoter is negatively correlated with up-regulation of *CD4* gene expression in the L7_2_ spleen at 21 dpi.

**Conclusions:**

The methylation fluctuation and mRNA expression change of *CD4* gene induced by MDV infection suggested a unique epigenetic mechanism existed in MD-susceptible chickens.

## Background

*CD4* encodes a glycoprotein, located on the surface of T helper (Th) cells and regulatory T cells. Through interaction with MHC class II molecules, CD4 directs the linage development of Th cells in immune organs and activates the CD4^+^ T cell maturation process [1]. Thus, the transcriptional level of *CD4* is directly related to T cell development [2]. In mice, *CD4* transcription is controlled by several *cis*-acting elements including enhancers, silencers and DNA methylation [3,4]. However, the epigenetic regulation of *CD4* gene in chicken and its relationship with any virus infection are still unclear.⋯

Marek’s disease (MD), a T cell lymphoma of chickens caused by the Marek’s disease virus (MDV), is characterized by mononuclear cell-infiltration in various organs including peripheral nerves, skin, muscle, and visceral organs [5], and is a worldwide problem for the poultry industry. A complex MDV life cycle was found in susceptible chickens during MD progression, which includes an early cytolytic phase (2-7 days post infection, dpi), latent phase (7-10 dpi), late cytolytic phase (from 18 dpi) and transformation phase (28 dpi and onwards) [6].

Epigenetics is the study of alterations that result in inherited changes in phenotypes despite the lack of DNA sequence polymorphisms and include DNA methylation, histone modification and chromatin remodeling [7]. It is described as the interaction between genes and environmental factors. Aberrant CpG methylation levels of the gene promoter region contribute to oncogenesis [8]. Viruses are one of the environmental agents that can cause alterations of DNA methylation level in host genes [9].

The focus of this study was to better understand the expression control of *CD4* by ascertaining the epigenetic status in the *CD4* promoter and the *CD4* expression in relation to MDV infection. Two inbred chicken lines, MD-resistant or –susceptible with the same MHC (major histocompatibility complex) haplotypes, from Avian Disease and Oncology laboratory (ADOL) were used [5]. We, therefore, measured the promoter methylation and transcription of the *CD4* gene before and after MDV infection of both lines. We found methylation alterations in the *CD4* promoter region after MDV infection differ between these two lines.

## Methods

### Animals, virus infection experiments and sample collection

USDA, Avian Disease and Oncology Laboratory (ADOL) chicken lines 6 (L6_3_^)^ and lines 7 (L7_2_^)^ chickens, which are MD-resistant and MD-susceptible, respectively, were obtained. For each line, the chickens were divided into two groups with 30 chickens infected by MDV and 30 uninfected controls. A very virulent plus strain of MDV (648A passage 40, VV+) was injected intra-abdominally on the fifth day after hatching with 500 plaque-forming units (PFU). Spleen samples were collected at 5 dpi, 10 dpi and 21 dpi, put in RNAlater (Qiagen, USA) immediately, and then stored at -80°C. All procedures followed the standard animal ethics and user guidelines.

### DNA extraction, bisulfite treatment and pyrosequencing

DNA was extracted from 20-30 mg spleen by NucleoSpin® Tissue Kits (Macherey-Nagel, Germany). 500 ng DNA was treated with sodium bisulfite and purified by EZ DNA Methylation-Gold Kit™ (ZYMO Research, USA). Primers for pyrosequencing were designed by PSQ Assay Design software (Biotage, Swedan) (Table 1). For cost reduction, a universal primer (5’-GGGACACCGCTGATCGTTTA-3’) was used in the PCR assays [10]. DNA methylation level analysis was performed with Pyro Q-CpG system (PyroMark ID, Biotage, Sweden) as previously described [10,11].

**Table 1:** Primers used in Pyrosequencing and quantitative PCR

Genes	Primers	***Sequence***	***Purpose***
*CD4*	F R Sequencing Assay	*5’- TTGAGATTATAYGTATTTGGAAGA -3’* *5’- GGGACACCGCTGATCGTTTA ACCTTTATATCTCCTCCTCTCCA -3’* *5’- AGTATTTATTGAGAGAAGTT -3’* *5’- **Y**GTAGATTGTAGTAGAGTTTGGAT**Y**G* *GTAGTAAGAT**Y**GTGTTGA**Y**GTTTT -3’*	*Pyrosequencing*
*GAPDH*	F R	*5’-GAGGGTAGTGAAGGCTGCTG-3’* *5’-ACCAGGAAACAAGCTTGACG-3’*	*quantitative PCR*
*CD4*	F R	*5’- TGTCAACGCCGGATGTATAA-3’* *5’- CTTGTCCATTGGCTCCTCTC-3’*	*quantitative PCR*

Y stands for C/T. Bold Y in the assay sequence is the CpG sites analyzed.

### RNA extraction and quantitative real-time RT-PCR

RNA from 30-50mg spleen was extracted using the RNAeasy Mini Kit (Qiagen, USA). Reverse transcription was carried out in 20 µl with 1 µg of total RNA by using SuperScript™ III Reverse Transcriptase (Invitrogen, USA) and oligo (dT)_12-18_ primers (Invitrogen, USA). Primers (Table 1) for quantitative real-time RT-PCR were designed by Primer3 online primer designer system (http://frodo.wi.mit.edu/). qPCR was performed on the iCycler iQ PCR system (Bio-Rad, USA) in a final volume of 20 µl using QuantiTect SYBR Green PCR Kit (Qiagen, USA) with the following procedure: denatured at 95^°^C for 15 min, followed by 40 cycles at 95 ^°^C for 30 s, 60 ^°^C for 30 s, 72 ^°^C for 30 s, then extended at 72 ^°^C for 10 min. Each reaction was replicated twice. The housekeeping gene *GAPDH* (glyceraldehyde-3-phosphate dehydrogenase) was used to normalize the assays.

### Statistical analysis

Promoter methylation levels and gene expression before and after MDV infection were compared by Student’s *t* test. An exact *F* test was performed to distinguish different methylation patterns [10]. Correlation between *CD4* DNA methylation and expression was tested by Pearson's correlation coefficient.

## Results

### *CD4* promoter methylation analysis before and after MDV infection

**Figure 1:**
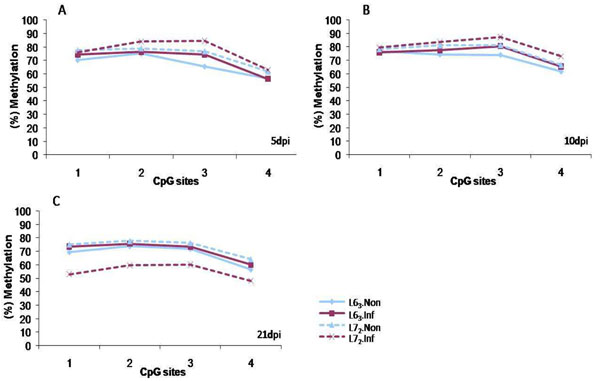
**CD4 promoter methylation levels at 5 (A), 10 (B) and 21dpi (C)**. Pyrosequencing result of the promoter methylation level of CD4 gene before and after MDV infection at different time points. A decrease of promoter methylation level was observed only L7_2_ chickens. 5dpi: 5 days post infection; 10dpi: 10 days post infection; 21dpi: 21 days post infection. L6_3_.Non: noninfected control of L6_3_ chicken; L6_3_.Inf: infected L6_3_ chicken; L7_2_.Non: noninfected control of L6_3_ chicken; L6_3_.Inf: infected L6_3_ chicken. n=4 for each line.

**Figure 2:**
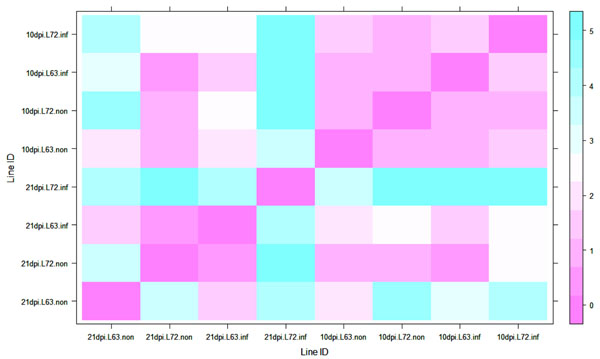
**Exact F test for DNA methylation patterns of CD4.** The methylation level of each of the CpG site in the promoter region of *CD4* gene was used to do an exact F test. *P* values matrix among L6_3_ and L7_2_ at 5, 10 and 21dpi. Color bar shows the significance level (*P* values with -log_10_(*P*). e.g., -log_10_(0.05) = 1.3; log10(0.01) = 2).

To determine the promoter methylation level of the *CD4* gene, a DNA sequence containing the CpG islands from the *CD4* gene promoter region (sequence shown in Table 1) was downloaded from UCSC (http://genome.ucsc.edu) and the methylation level was determined by pyrosequencing. The CpGs in the promoter of *CD4* exhibits a high (>70%) methylation level in both L6_3_ and L7_2_ chickens before MDV infection. During MD progression, no significant methylation changes of *CD4* promoter were detected in L6_3_ chickens at 5, 10 and 21 dpi or in L7_2_ chickens at 5 and 10 dpi (*P*>0.05, Figure 1, and Figure 1A and 1B); however, the significant down-regulation of *CD4* promoter methylation level was observed at 21 dpi in L7_2_ chickens (*P*< 0.05, Figure 1C). The result from the exact *F* test revealed that the *CD4* promoter methylation pattern in L7_2_ infected samples at 21 dpi was significantly different from any other groups (Figure 2).

### *CD4* gene expression at 21 dpi

**Figure 3:**
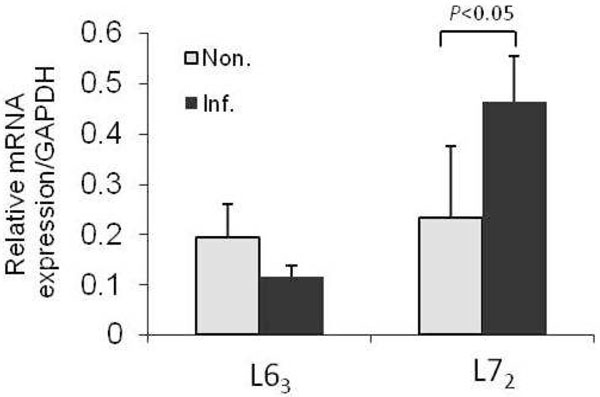
**Relative mRNA expression of CD4 gene at 21dpi**. Real-time quantitative PCR was used to detect the mRNA expression level of *CD4* gene in different chicken lines with or without MDV infection at 21dpi. The relative expression level of *CD4* gene was normalized to a house keeping gene *GAPDH*. Non.: noninfected control samples; Inf.: infected samples. n=4 for each line and treatment.

**Figure 4:**
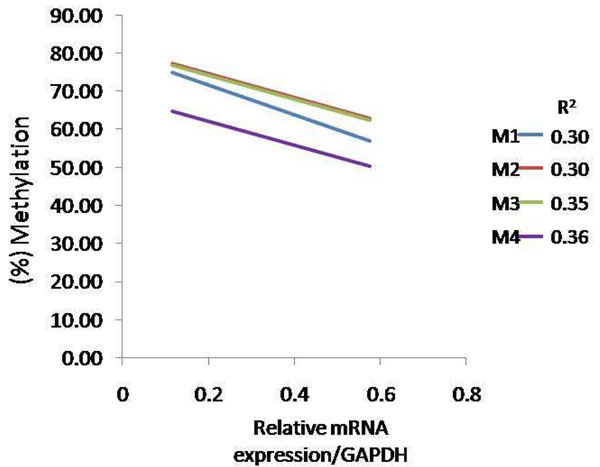
**Correlation between CD4 promoter methylation and relative gene expression in L72 spleen at 21dpi**. Correlation analysis of the relationship between methylation and CD4 gene mRNA expression level. R represents for the correlation coefficient. M1: CpG site 1; M2: CpG site 2; M3: CpG site3; M4: CpG site 4. n=4 for each line and treatment.

To ascertain if the *CD4* gene transcription level is influenced by its promoter methylation changes at 21 dpi, we conducted quantitative PCR. We found a significantly higher expression of *CD4* gene in L7_2_ infected samples compared with noninfected control samples (*P*<0.05) (Figure 3), whereas no significant up or down-regulation of *CD4* expression was detected in L6_3_ chickens after MDV infection (P>0.05). Hereinafter, further correlation analysis showed that methylation level of all the detected CpG sites existed a negatively relationship with *CD4* gene expression in L7_2_ chicken at 21dpi (Figure 4).

## Discussion

The *CD4* gene and its regulatory sequences are conserved [12]. In human and mouse, multiple protein or transcription factor binding sites, including the Myb binding site, Elf-1 binding site, and Ikaros binding site, were found in the promoter region of *CD4*, which is involved in the on/off switching of *CD4* gene expression [4]. These regulatory sites were also found in the chicken *CD4* promoter with potential functions in its expression [12]. It is well known that epigenetic factors such as DNA methylation and histone modifications play important roles in transcriptional regulation in mammals [7]. For example. the methylation change in at least one CpG site of *CD4* gene in mouse is related to CD4^+^ T cell differentiation [3]. In this study, we thus examined the methylation status in the promoter region of *CD4* gene in chickens related to MDV infection.

MDV is an oncovirus using CD4+ T cell as a target for latent infection and transformation, which may have interactions with the CD4 gene at the epigenetic level [13]. In our previously study, two mutations (CG→TG) were identified in the DNMT3b gene between L63 and L72 chickens [10], which implied that the DNA methylation machinery may be different in the two lines in response to MDV infection. In this study, the methylation levels on the promoter region of the CD4 gene were fluctuated over different time points of MDV infection in MD-susceptible chickens, especially during the late cytolytic phase. The quantitative PCR results confirmed that CD4 expression in L72 chicken during the late stages of MDV infection was upregulated while the CD4 promoter methylation was down-regulation. Since the expression of CD4 is essential for CD4+ T cell development and activation, it may suggest that there are different epigenetic machineries of activation of CD4+ T cells by MDV infection through regulation of CD4 methylation levels between MD-resistant and susceptible chicken lines. From previous studies, it was found that the number of infected CD4+ T cells were similar during the early phase (cytolytic phase) of MDV infection between MD-resistant and –susceptible chicken lines, but was increased during cytolytic phase in MD-susceptible chicken line and decreased in MD-resistant chicken line [14]. Additionally, in MD-resistant chicken line, CD4+ T cell is latently infected, but cannot be transformed, whereas in MD-susceptible chicken lines the infected CD4+ T cell can be transformed after the latent phase [5,15]. Taken together, the methylation change of CD4 gene gives us an important clue that epigenetic alteration could associate with MD etiology. Therefore, future efforts will disclose the epigenetic landscapes, including genome-wide DNA methyltion and histone modifications, in immune organs and specific cell types, such as the CD4+ T cell, which will supply rich information to explore the epigenetic machinery related to chemical and physiological mechanisms of MD resistance or susceptibility.

## Conclusions

In conclusion, the methylation fluctuation and mRNA expression of *CD4* gene induced by MDV infection suggested a unique epigenetic mechanism existed in MD-susceptible chickens.

## Competing interests

The authors declare that they have no competing interests.

## Authors' contributions

JL performed the experiments and prepare the manuscript. YY and FT performed the experiments. HMZ and SC designed and performed the viral challenge experiment. JZS designed and wrote the paper.
